# Arsenicum-album

**Published:** 1895-11

**Authors:** 


### HOMŒOPATHIC PHYSICIAN,

A MONTHLY JOURNAL OF

HOMŒOPATHIC MATERIA MEDICA AND CLINICAL MEDICINE.

"If our school ever gives up the strict inductive method of Hahnemann, we are lost, and deserve only to be mentioned as a caricature in the history of medicine."—CONSTANTINE HERING.

Vol. XV. NOVEMBER, 1895. No. 11.

### EDITORIAL.

ARSENICUM-ALBUM.—Very little can be said of the symptoms of the abdomen, as Dr. Lippe gave but few notes and they were not important.

Proceeding now to the symptoms relating to the rectum and stool, we find that, as was stated in last month's article, the stool is loose and accompanied with vomiting. A characteristic of the stool is that it is acrid and it burns as it passes the border of the anus, and there is tenesmus. The diarrhœa of Arsenicum is worse from eating and drinking. The diarrhœa is accompanied with dragging pain around the umbilicus. Arsenic has bloody stools and so also have Phosphorus, Hamamelis, Alumina, and Millefolium. Arsenicum has prolapsus of the rectum, and the rectum remains protruded after hemorrhage of the varices of the lining membrane. Arsenicum has hemorrhoids that burn like fire; with amelioration by lying down. The hemorrhoids pain as if from hot needles. This is a characteristic of Arsenicum. Thus we find headache under this remedy as if a hot wire were thrust into the ramifications of fifth pair of nerves. We find, also, swollen upper lip with pain as if from red-hot needles. Pains in the face generally as if from red-hot needles. This statement thus fixes in the mind the association of the drug Arsenicum with the symptom, pain as if from red-hot needles or wires. The pain of the hemorrhoids is worse from walking or sitting, but not during stool, and, as previously stated, better from lying down. Apis has hemorrhoids with burning, stinging pains. See May number of this journal, page 203, line five from the top.

Arsenicum-album has diarrhœa from taking anything cold into the stomach. Therefore it has diarrhœa from drinking cold water. In connection with this symptom we may add the following notes:

Capsicum, as soon as he drinks he must go to stool, but passes only a little mucus.

Argentum-nitricum, water seems to run right through him.

Capsicum, drinking water causes chills with shuddering, tenesmus, and purging, the stools being thin.

Arsenicum, Argentum-nitricum, Cina, Croton-tiglium, and Podophyllum have diarrhœa after drinking water.

Under Arsenicum we find diarrhœa of thin, dark-brown or greenish mucous stools smelling like old putrid pus.

After stool the colic of Arsenic ceases.

Arsenicum has restlessness and pain in the bowels, followed by a discharge from the rectum of a black fluid which burns like fire as it passes through the anus.

Arsenicum has discharge of prostatic fluid during passage of loose stool.

These stool symptoms of Arsenicum naturally suggest a great number of comparisons with other remedies. These comparisons are so numerous one would hardly know where to stop. It would in effect be equivalent to writing a treatise on diarrhœa. But this has already been done by Dr. James B. Bell, of Boston. His masterpiece could not be equalled, much less excelled, and so no attempt in that direction will be made in these pages. We can only advise any reader who from any cause may not have provided himself with a copy of this monograph to do so without delay.

Moreover, the whole intent of these comments, as was stated at the beginning, is simply to give to the profession some dim idea of the nature of the lectures upon materia medica delivered by the late Dr. Adolph Lippe when he was at the zenith of his success as a homœopathic prescriber. Consequently the notes have been confined to his statements, the occasional additions of the editor being clearly stated to avoid misapprehension as to their authority.

